# Better Alone or in Ill Company? The Effect of Migration and Inter-Species Comingling on *Fascioloides magna* Infection in Elk

**DOI:** 10.1371/journal.pone.0159319

**Published:** 2016-07-27

**Authors:** Mathieu Pruvot, Manigandan Lejeune, Susan Kutz, Wendy Hutchins, Marco Musiani, Alessandro Massolo, Karin Orsel

**Affiliations:** 1 Wildlife Health and Health Policy Program, Wildlife Conservation Society (WCS), Phnom Penh, Cambodia; 2 Canadian Wildlife Health Cooperative (CWHC), Calgary, Alberta, Canada; 3 Department of Ecosystem and Public Health, Faculty of Veterinary Medicine University of Calgary (UCVM), Calgary, Alberta, Canada; 4 Department of Microbiology and Infectious Diseases, Faculty of Medicine, University of Calgary, Calgary, Alberta, Canada; 5 Faculty of Environmental Design, University of Calgary, Calgary, Alberta, Canada; 6 Department of Production Animal Health, Faculty of Veterinary Medicine University of Calgary (UCVM), Calgary, Alberta, Canada; INIAV, I.P.- National Institute of Agriculture and Veterinary Research, PORTUGAL

## Abstract

Migratory movements and alteration of host communities through livestock production are examples of ecological processes that may have consequences on wildlife pathogens. We studied the effect of co-grazing of cattle and wild elk, and of elk migratory behaviour on the occurrence of the giant liver fluke, *Fascioloides magna*, in elk. Migratory elk and elk herds with a higher proportion of migratory individuals were significantly less likely to be infected with *F*. *magna*. This may indicate a decreased risk of infection for migratory individuals, known as the “migratory escape” hypothesis. Elk herds overlapping with higher cattle densities also had a lower prevalence of this parasite, even after adjustment for landscape and climate variables known to influence its life cycle. Serological evidence suggests that even in low-prevalence areas, *F*. *magna* is circulating in both elk and cattle. Cattle are “dead-end” hosts for *F*. *magna*, and this may, therefore, indicate a dilution effect where cattle and elk are co-grazing. Migratory behaviour and host community composition have significant effects on the dynamics of this wildlife parasite; emphasizing the potential impacts of decisions regarding the management of migratory corridors and livestock-wildlife interface.

## Introduction

In the foothills of the Rocky Mountains in Alberta, Canada, 500,000 hectares of rangeland are used for beef cattle grazing, resulting in a significant overlap between cattle and the North American elk (*Cervus elaphus*) [1]. Other areas protected under federal or provincial regulations, however, do not allow livestock grazing. These variations in land-use and land access result in significant differences in host community compositions, an important driver of the dynamics of infectious diseases [2–4]. *Fascioloides magna*, also known as Giant liver fluke, is commonly found in elk and deer (*Odocoileus virginianus* and *Odocoileus hemionus*), the definitive hosts of this trematode [5–6]. The adult form, located in the liver, excretes eggs that are released via the host faeces, and the subsequent larval stages require the presence of aquatic snail in the environment for their development [7].

*Fascioloides magna* was reported to spill over from elk and deer to cattle [8–9], however, cattle are dead-end hosts that rarely excrete eggs in their faeces, as worms and eggs are contained in thick-walled cysts, preventing the infection to become patent [6–7,10]. It is not known how the sympatry with this dead-end host (i.e. cattle) may affect the prevalence of *F*. *magna* in free-ranging elk, and in particular, if the presence of cattle may exert a dilution effect and reduce the prevalence in elk, as reported in other multi-host systems [3, 11–14].

Furthermore, elk can adopt either a migratory (seasonal migration between clearly distinct winter range and summer range), resident (year round in the same location) or dispersal behaviour (movement beyond a normal range with no return to area of origin), sometimes within the same herd [15–16]. Migration is thought to influence pathogen transmission dynamics in free-ranging wildlife [17–20]. In particular, the migratory escape hypothesis suggests that moving away from contaminated habitat may prevent infection in migratory individuals and decrease prevalence in migratory populations [20–21]. Environmental characteristics and climate conditions have a strong influence on the availability of intermediate hosts and the development and survival of *F*. *magna* [7]. The presence of standing or flowing water influence the distribution of aquatic snails and their activity is driven by seasonal temperature changes and frost-free periods. Vegetation, water availability and weather conditions also affects the survival of free-living aquatic larvae, and temperatures affect the period and duration of larval stage development. [7, 22]. As a result, the abundance of free-living infective stages varies seasonally [7], and migratory elk may “escape” contaminated habitats during the peak infection period, thereby reducing their risk of infection.

In this study, we tested the two hypotheses of a dilution effect and ‘migratory escape’ for *F*. *magna* in this multi-host system. We hypothesize that where elk and cattle live in sympatry, a dilution effect would reduce the risk of *F*. *magna* infection in elk. We would, therefore, expect (i) lower prevalence of *F magna* infection in elk herds comingling with cattle than in elk herds segregated from cattle, and (ii) serological evidence of *F*. *magna* exposure in cattle living in sympatry with elk. We also hypothesize that (iii) migratory individuals would have a lower risk of being infected with *F*. *magna*, (iv) egg counts of infected individuals would be negatively associated with migration distance, and (v) herd prevalence would be lower in herds with higher proportion of migratory individuals. To test these hypotheses, we used a comprehensive eco-epidemiological approach by drawing from parasitological, serological and statistical evidences, in particular through the development of serological assays applied to both elk and cattle, and the spatial analysis of elk telemetry and remote sensing data.

## Material and Methods

### Ethic statement

Elk capture protocols carried out by the Montane Elk Research Program were approved by the Universities of Alberta and Calgary and the government of Alberta (Permit Numbers: BI-2008-19, RC-06SW-001 and 23181CN). Sample and data collection in cattle ranches, as well as the elk faecal sample collection, was reviewed and approved by the University of Calgary Conjoint Faculties Research Ethics Board (file no. 6598), and the University of Calgary Certification of Animal Protocol Approval M09123. All animal use followed the guidelines established by the Canadian Council on Animal Care. Additionally, elk faecal sample collection procedures were reviewed and approved for obtention of field research permits and land access: Parks Canada research permit JNP-2010-4432; Alberta Parks Research and collection permit 10–007; Alberta Sustainable Resource Development Collection License #40820 and Research permit #40799. Access to private land was also individually requested to each landowner.

### Elk herds and study area

**Fig 1:**
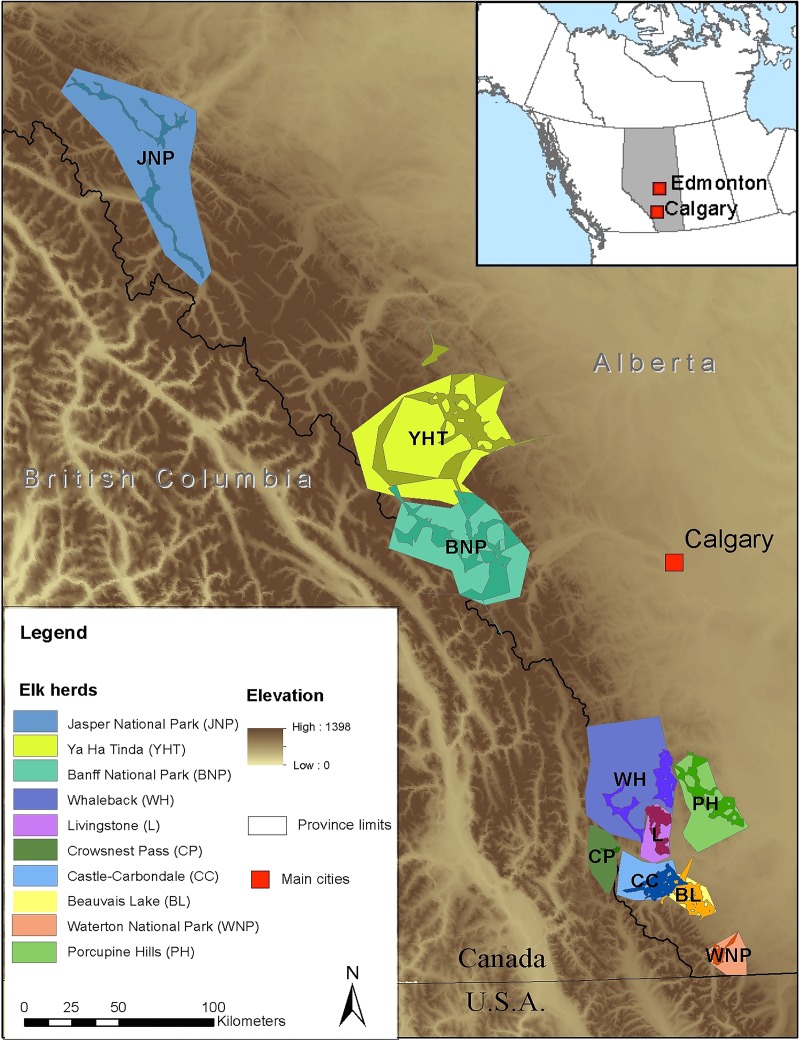
Location and home range estimate of the ten elk herds in western Alberta, Canada. The light-colored polygons are approximate delimitation of each herd home range, the dark-colored polygons are the resident elk home range of each herd estimated by adaptative local convex hull.

Ten elk herds in western Alberta were included in this study (Fig 1). Five of these herds, namely Castle-Carbondale (CC), Beauvais Lake (BL), Livingstone (L), Whaleback (WH) and Porcupine Hills (PH), were in areas of high cattle density (‘exposed’ herds), while the other five, namely Jasper National Park (JNP), Ya Ha Tinda (YHT), Banff National Park (BNP), Crowsnest Pass (CP), Waterton National Park (WNP), had no or limited contact with cattle (‘non-exposed herds) (Table 1). Most herds included both migratory and resident individuals. Migratory and resident elk home ranges typically overlap in the winter but migratory animals move westward to areas of higher elevation in the summer [16,23–24]. Exposed elk herds overlapped with cattle in both winter and summer, with their winter range dominated by private land, and their summer range expanding on public land, including public grazing allotments.

**Table 1:** Descriptive statistics of the ten elk herds in Alberta, Canada.

Herd (abbreviation)	Exposure to cattle	Estimated herd size	Estimated cattle density (in beef cows /km^2^)^d^	Estimated proportion of the herd with migratory behavior^g^	Captured elk			Additional faecal sample collection
					Project	Serum samples	Faecal samples at capture	
Beauvais Lake (BL)	Exposed	150–250^e^	8.04	57	Montane Elk Research Program	9	8	69
Castle-Carbondale (CC)		500–700^e^	8.04	93	Montane Elk Research Program	72	34	80
Livingstone (L)		340^e^	7.85	95	Montane Elk Research Program	16	12	74
Porcupine Hills (PH)		450–700^e^	9.12	50	Montane Elk Research Program	8	3	69
Whaleback (WH)		700–1000^e^	3.17	87	Montane Elk Research Program	30	12	93
Waterton (WNP)^a^	Non-exposed	900^e^	1.93	20	Montane Elk Research Program	16	10	64
Crowsnest Pass (CP)^b^		200^e^	0.18	95	Montane Elk Research Program	17	7	38
Jasper National Park (JNP)^c^		1300^f^	0.08	0	Parks Canada	31	0	55
Banff National Park (BNP)^c^		215^f^	0.05	0	Parks Canada	20	0	16^h^
Ya Ha Tinda (YHT)		1000^f^	1.55	49	University of Alberta—University of Montana	80	0	70

^a^. The elk herd only partially spread outside the park boundaries during calving season

^b^. Cattle are only present for summer public grazing

^c^. JNP and BNP are resident herds of the national parks and have no interaction with cattle.

^d^. Source: Statistics Canada—Agricultural census 2011

^e^. Source: Alberta Conservation Association Winter Survey 2001–2002 and 2006

^f^. Source: Parks Canada

^g^. Proportion for BL, CC, L, PH, WH, WNP and CP were estimated from the collared animals; for JNP and BNP, Parks Canada’s projects were especially focused on resident individuals of Banff and Jasper town-sites; for YHT see [16]

^h^. Opportunistically obtained from road-killed elk, culling of nuisance animals, or captured animals.

### Montane Elk Research Program elk dataset

Over 170 elk from 7 herds (CC, BL, CP, L, WH, WNP and PH) in the Montane Elk Research Program (www.montaneelk.com) (hereafter referred to as MERP elk) were captured and fitted with Global Positioning System (GPS) telemetry collars (Lotek 4400, 3300, 2200 series and ArgosTrack, and Telonics TGW-3600) from 2007 to 2013 winter seasons using helicopter net-gunning,. For each individual, the telemetry data were visually inspected to classify the animal behaviour as migratory, resident or dispersal, and proportions of migratory individuals in each herd calculated (Table 1). Individual home ranges were estimated using the non-parametric adaptative local convex hull algorithm implemented in the LoCoH R script [25], with a = 40000 as defined by preliminary testing according to [25].

The age of MERP elk was determined by cementum analysis of the upper canine extracted at capture (Matson's Laboratory, MT, USA). Blood and faecal samples were collected from these captured individuals (Table 1). Serum was separated after centrifugation and stored at -20°C. Faecal samples were stored at -80°C. All MERP elk sera were tested by ELISA based on the full worm antigen (FWA) and results were expressed as a sample-to-positive ratio (S/P ratio) based on a positive and negative reference samples (see below).

### Faecal sample dataset

A limited number of faecal samples (n = 86) could be obtained from the MERP elk (captured individuals) from seven herds only (Table 1). Therefore, to increase the number of herds and the overall sample size, and to allow assessing the prevalence of egg-shedding elk, we conducted a parasitological survey in the seven aforementioned herds, and three additional herds non-exposed to cattle (YHT, JNP, and BNP). Fresh faecal samples were collected from distinct individuals from the different sub-groups accessible in each herd as described in [1] and in S1 File (hereafter referred to as ‘Faecal sample dataset’; see sample size in Table 1).

Two grams of each elk faecal sample were processed using a sedimentation technique commercialized as Flukefinder^®^ (Visual Difference, Moscow, Idaho, USA), according to manufacturer instructions. The Flukefinder^®^ consists of a two sieve system with different pore size. The first sieve isolates the larger faecal debris while the second retains the *F*. *magna* eggs. Eggs were recovered by back-flushing this second sieve with water, sedimenting this solution twice for 2 minutes each, and then pouring the remaining 3 mL of sediment into a petri dish. Petri dishes were examined under dissecting microscope at a 50X maximum magnification and eggs were counted.

Telemetry data was then used to estimate home ranges at the herd level. In addition to the MERP elk GPS locations, telemetry data was also obtained from the Ya Ha Tinda elk herd (n = 220 collared elk) [16], and from elk in the resident herds of Banff (n = 628 collared elk) and Jasper National Parks (n = 8 collared elk) [26–29].

The telemetry data was then split by herd, by migratory behavior, and by season for migratory elk in order to obtain the resident herd locations, the summer range locations and the winter range locations of each herd; dispersal elk were excluded. For each data subset, the home ranges were estimated as previously described. These home ranges are therefore describing the herd (and not distinct individuals as in the MERP elk dataset).

### Cattle sample dataset

The dilution effect relies on the fact that a non-competent host ingests *F*. *magna* metacercariae that then cannot complete their development. Thus, a dilution effect would be very unlikely if cattle were not found to be exposed to *F magna* in this area. To test the exposure of cattle to the giant liver fluke, fifteen cow-calf operations overlapping with the MERP elk herds and containing more than 100 cows were sampled, as described more extensively in [30]. In each ranch, blood samples from 30 cows over 2 years old were collected from the coccygeal vein by systematic sampling (1 sample every *N* cows, with *N = total number of cows meeting the selection criteria / 30*, the first cow randomly picked among the *N* first cows using a random number table) during handling operations. Age, breed and origin (raised on ranch or purchased) of the sampled cows were recorded. Blood was later centrifuged and serum was separated and stored at -20°C.

### Serology development and testing

Because infected cattle are dead-end hosts and usually do not shed eggs, ante-mortem diagnosis of *F*. *magna* relies on serological tests and/or clinical signs. We developed and validated a western blot (WB) and enzyme linked immuno-sorbent assay (ELISA) in elk and cattle, and used these diagnostic tools to detect non-patent infections in both species. A full description of essay development protocols and test validation can be found in S2 File.

All MERP elk sera were tested by ELISA: for each well, the optic density (OD) value was re-expressed as a sample-to-positive ratio (S/P ratio = [OD_Sample_-OD_Neg_]/[OD_Pos_-OD_Neg_]) based on a positive and negative reference samples selected during preliminary testing and consistently used across all plates. Samples were then classified as positive or negative based on the cut-off values defined for this test (S2 File).

For cattle serum samples, the ELISA was used as a screening test and only samples with S/P ratios higher than 0.65 (using a positive serum sample from previously confirmed infected cow as reference) were processed in WB. To ensure the screening procedure had an appropriate sensitivity, we additionally tested 30 samples with S/P ratios lower than 0.65.

### Habitat characterisation

Habitat was characterised over the individual home range of the MERP elk (MERP elk dataset) and over the herd-level home range (faecal sample dataset), to use in multivariable analysis and ensure that the differences of *F*. *magna* prevalence observed between migratory behaviour or cattle exposures were not related to differences of environment between individuals or herds (confounding effect; see Statistical Analysis section) [31]. However, collinearity is a frequently encountered problem when trying to describe the environment in ecological or epidemiological studies [32]. Principal component analysis (PCA) is an effective method to reduce the number of habitat variables and the correlations between these variables [32]. A 1km grid was overlaid on the study area (defined as a convex hull around all herd home ranges). For each grid cell, summary statistics of the environment and climate characteristics were computed as described in Table 2, and included in the PCA (excluding the cattle density). Principal components were rotated to facilitate interpretation using the equamax method [33]. Eigenvalues above 1 were retained in the PCA. The Keiser-Mayer-Olkin (KMO) index was optimized by removing variable with lower communality [33]. The Anderson-Rubin method for score calculation was used to obtain orthogonal (i.e., independent) factor scores. A 1km-resolution raster was built for each PCA factor, and the mean value of these PCA factors was then extracted for the home range of each individual MERP elk (MERP elk dataset) and of each herd (Faecal sample dataset).

**Table 2:** Data source and handling of variables used to describe the elk herds in multivariate analysis.

Variable	Data Type	Description	Source	Summary statistics^a^
Snow cover	Raster (continuous)	Snow cover in % (1 km resolution)	NCEP/NCAR Reanalysis Project (at the NOAA/ESRL Physical Sciences Division)	Mean for winter period, Mean for summer period
Wind speed	Raster (continuous)	Wind speed in m/s (1 km resolution)		Mean for winter period, Mean for summer period
Air temperature	Raster (continuous)	Air temperature in K (1 km resolution)		Mean for winter period, Mean for summer period
Precipitation	Raster (continuous)	Precipitation in kg/m^2^ (1 km resolution)		Mean for winter period, Mean for summer period
Surface runoff	Raster (continuous)	Surface runoff water in kg/m^2^ (1 km resolution)		Mean for winter period, Mean for summer period
Soil moisture	Raster (continuous)	Soil moisture in kg/m^2^ (1 km resolution)		Mean for winter period, Mean for summer period
Cattle density	Polygon	Cattle density in beef cow /km^2^	Agriculture Canada– 2006 census; Agriculture Census of the United States—2007	Area-weighted average of beef density of districts overlapping the home range
Water	Polyline	Delimitation of streams, creek, river, lakes and ponds contours.	National Hydrographic Network—obtained from GeoBase	Cumulative length of each type of water source
Normalized Difference Vegetation Index (NDVI)	Raster (continuous)	30m resolution	MODIS/Terra Vegetation Indices 16-Day L3 Global 250m SIN Grid. NASA Land Processes Distributed Active Archive Center (LP DAAC).	Mean over home range
Land cover	Raster (categorical)	16 categories vegetation cover data (30m resolution)	[34]	Proportion of home range covered by each land type
Elevation, Slope	Raster (continuous)	Digital Eleveation Model (30m resolution)	DMTI Spatial via University of Calgary	Mean over home range

^a^ Winter period: Nov 15th–Apr 15th (averaged over 2007 to 2011) Summer period: Apr 15th–Nov 15th (averaged over 2007 to 2011)

### Statistical analysis

#### MERP elk dataset

On the MERP elk dataset, the associations between the *F*. *magna* ELISA results and the cattle density on one hand, and the elk migratory behavior on the other hand were assessed with multi-level logistic regression models with the herd as random effect. Additionally, the travel distance between winter and summer range was assessed by computing for each elk the longest distance within the minimum convex polygon obtained from its telemetry data. The association of this travel distance with the elk ELISA S/P ratio and faecal egg count was assessed in a mixed-effect regression and multi-level negative binomial regression respectively, with herd as a random effect to adjust for herd clustering.

To control for the potential confounding effect of environmental variables (that is, to ensure that the observed effects of cattle density and migratory phenotype are not related to differences of environmental variables between elk home ranges), these associations were reassessed after including in the regression models the composite variables produced by the PCA. Multi-level logistic regression models, based on the outcome of the ELISA test (with the cut-off value optimizing both sensitivity and specificity), were fitted with the R package lme4 [35]. Age, sex, migratory behaviour, elk density, the six PCA factors (the factor mainly related to wind speed was excluded as judged less relevant and not associated to the outcome in univariate analysis), and the cattle density were the variables in the full model (with herd as a random effect). We computed models with all possible combinations of these variables in the R package MuMIn [36] and ranked them by corrected Akaike Information Criteria (AICc) to select the most parsimonious model best supported by the data. To assess the predictive ability of the best model, we computed the area under the ROC curve (AUC) and estimated the average proportion of correctly predicted outcomes in a 10-fold cross-validation. To appropriately incorporate the model selection uncertainty in the estimate of cattle density effect, model averaging was carried out as a weighted mean of regression coefficients (based on Akaike weights) [37]. A similar approach was used with a mixed-effect regression on the continuous outcome (S/P ratio) of the ELISA.

#### Faecal sample dataset

On the faecal sample dataset, the association between the overlap of elk with cattle (as binary variable) and the individual coproscopic results was assessed by a *χ*^*2*^ test. The effect of the average cattle density within the elk herd home range (based on the Agriculture Canada census data, Table 2), or of the proportion of migratory individuals, on the *F*. *magna* coproscopy prevalence, were assessed in binomial logistic regression models.

Relevant variables included the beef cattle density and the proportion of migratory elk in the herd (our main two predictors of interest), elk density, and the habitat PCA factors. Because the number of herds was limited, it was not possible to include all the herd-level variables. Models with all combinations of three of these variables were compared by the AICc, and the averaged model was computed as previously described. Because, with the field-collected faecal samples (faecal sample dataset), it was not possible to determine if a sample was from a migratory or resident elk, we used the habitat characteristics over the resident home range for each of the herds (thus assuming that all samples were collected from resident elk). To test the sensitivity of our model to this assumption, we also extracted the habitat characteristics for the migratory home ranges and compared the outcome of the multivariate models under this alternative assumption: we did not observe any substantial difference in the model outcomes.

## Results

### Elk coproscopic and serological results

The apparent prevalence based on coproscopic examination, adjusted for herd size and sampling weight of each herd, was 32% (95% CI [29, 35]) from the field-collected faecal samples, and 22% (95% CI [14, 32]) from the MERP elk faecal samples. The prevalence for the MERP elk tested by ELISA was 23% (95% CI [16, 30]) with the 0.9 cut-off maximizing specificity (S2 File), and 31% (95% CI [23, 40]) with the 0.71 cut-off optimizing both sensitivity and specificity. However, there was a high variability of prevalence among elk herds (Table 3). With the 0.9 cut-off value, five elk were seropositive for *F*. *magna* in 4 of the 5 elk herds with low or null proportions of faecal-positive, while 12 were seropositive from these 5 herds when the 0.71 cut-off value was used (Table 3). Females had higher odds of being seropositive than males after adjustment for herd clustering (OR = 3.9, 95% CI [1.1, 16.8]).

**Table 3:** Coproscopy and ELISA results in 10 elk herds.

Herd	BL	CC	L	PH	WH	WNP	CP	JNP	BNP	YHT
Number tested by ELISA	9	70	13	6	27	16	15	*nt*	19	*nt*
Positive at SP ratio>0.9	1	2	0	1	1	10	3	*nt*	18	*nt*
Proportion (in %) of S/P ratio>0.9	11±21	2.9±3.7	0	17±33	3.7±7.1	63±24	20±20	*nt*	95±10	*nt*
Positive at SP ratio>0.7	1	5	1	3	2	11	4	*nt*	19	*nt*
Proportion (in %) of S/P ratio>0.7	11±21	7.1±5.7	7.7±14.8	50±44	7.4±9.9	69±23	27±22	*nt*	100	*nt*
Number faecal sample	77	118	86	73	105	74	45	55	16	70
Positive by Flukefinder	2	0	0	0	0	53	2	39	12	19
Proportion (in %) faecal-positive	2.6±3.5	0	0	0	0	72±10	4.4±6.1	71±12	75±22	27±10

CC = Castle-Carbondale, BL = Beauvais Lake, L = Livingstone, WH = Whaleback and PH = Porcupine Hills are in area of high cattle density; JNP = Jasper National Park, YHT = Yaha Tinda, BNP = Banff National Park, CP = Crowsnest Pass, WNP = Waterton National Park are in low cattle density areas; *nt* = not tested.

### *F*. *magna* serology in cattle

Among the 57 cattle sera with ELISA S/P ratio above 0.65 and tested in western blot, 20 were WB-positive, while the 30 below 0.65 were all WB-negative. Sixteen of the 20 WB-positive samples were from one ranch seasonally exposed to elk from the WNP herd, in which 53% of cows were seropositive and clinical cases were previously reported in calves of 6 month to a year of age. The four other seropositive cows were from ranches overlapping with L, WH, CC, BL elk herds. All the cows that were positive by WB and ELISA had been raised on the ranch (i.e. not been imported from other areas).

### Habitat characterisation

The PCA summarized the landscape and climate data in 7 orthogonal factors explaining 75% of the variance of the data (Table 4).

**Table 4:** PCA factor description.

PCA factors	Contributing factors^a^	Interpretation
1	0.846*Precipitation + 0.865*Soil moisture + 0.805*Surface runoff	Surface water
2	0.757*Elevation—0.658*Agriculture land—0.729*Air temperature	High elevation landscape
3	0.709*NDVI + 0.644*Dense Coniferous Forests + 0.790*Moderate Coniferous Forests	Sub-Alpine landscape
4	0.833*Mixed forest + 0.787*Broad leaf forest—0.446*Elevation	Montane landscape
5	0.983*Wind speed	Wind speed
6	0.657*Length of water streams + 0.688*Open coniferous forest + 0.428*Herbaceous	Running water (Montane landscape)
7	0.705*Mid-size lake shore + 0.726*Pond shore	Standing water

^a^Indicates the 3 variables with highest contribution to the factor (variables with coefficients <0.3 were not included in the table)

### Effect of cattle density and elk migration

#### MERP elk dataset

In the MERP elk dataset, migratory individuals had lower odds of being seropositive for *F*. *magna* (OR = 0.25, 95% CI [0.06, 0.99]). Additionally, the ELISA S/P ratio and the faecal egg counts were negatively associated with the longest distance of each elk minimum convex polygons (in kilometers) in mixed-effect linear (β = -0.004, 95% CI [-0.006, -0.001], p = 0.009) and mixed-effect negative binomial (β = -0.10, 95% CI [-0.19, -0.02], p = 0.018) regressions, respectively. The beef cattle density within the home range of each elk was not significantly associated with the *F*. *magna* coproscopic or serological results.

The multi-model average of the multi-level logistic regression indicated the higher relative importance of the migratory behavior and beef cattle density (negatively associated), and age and PCA factor 7 (positively associated) (Table 5). Migratory status, age and beef cattle density on the elk home range were also the 3 variables included in the best model (Table 6). However, only migration was significantly associated in the best model.

**Table 5:** Multi-model averages of the four modelling approaches used on the two datasets.

Dataset	Faecal samples dataset				MERP elk dataset							
Model	Population-averaged binomial regression				Multi-level logistic regression				Multi-level regression (on ELISA SP ratio)			
Variables	Rank^a^	w_+_^b^	β^c^	S.E.^d^	Rank	w+	β	S.E.	Rank	w+	β	S.E.
Factor 7	**1**	0.65	2.29	0.99**	**3**	0.52	2.06	1.28	**2**	0.49	0.26	0.12**
Cattle density	**3**	0.49	-1.45	0.43**	**4**	0.44	-0.57	0.45	**4**	0.18	-0.09	0.05*
Factor 6	**4**	0.46	-3.20	0.80**	6	0.29	0.90	1.64	7	0.13	0.05	0.17
Migratory	5	0.43	-3.40	1.17**	**1**	1.00	-1.45	0.82*	**1**	1.00	-0.23	0.09**
Factor 2	**2**	0.56	2.17	1.10*	7	0.28	0.16	2.22	5	0.16	0.09	0.20
Factor 4	7	0.07	0.73	0.77	8	0.27	-0.24	1.08	9	0.07	0.01	0.09
Factor 1	8	0.03	-0.04	1.75	6	0.29	1.34	2.24	6	0.15	-0.01	0.22
Factor 3	9	0.02	2.10	2.22	6	0.29	-0.82	1.33	8	0.09	0.05	0.11
Elk density	6	0.24	33.28	19.58	7	0.28	0.11	0.22	10	0.03	0.02	0.02
Age	na	na	na	na	**2**	0.55	0.07	0.07	11	0.01	0.01	0.01
Sex	na	na	na	na	5	0.32	0.74	0.96	**3**	0.23	0.12	0.07*

^a^Rank according to variable relative importance = sum of Akaike weights of models containing the variable

^b^Variable relative importance = Cumulative Akaike weights of variables (sum of Akaike weights of models containing the variable); the first 4 ranks are bolded

^c^Multi-model averaged regression coefficients (weighted by the Akaike weight of each model)

^d^Standard Errors of regression coefficients (adjusted for model selection uncertainty)

** p-value <0.05

* p-value <0.1

**Table 6:** Best models according to Akaike Information Criterion corrected for small sample bias (AICc).

Dataset	Model^a^	Best model^b^	K^c^	logLik^c^	AICc	Adjusted R^2^ (%)	CV^c^ (%)
Faecal sample dataset	Binomial logistic	Migratory** + Factor 2** + Factor 7**	4	-14	44.1	na	na
MERP elk dataset	ML logistic	Migratory** + age + CattleDensity* + (herd)^d^	5	-42	94.8	56.2	87.3
	ML regression	Migratory** + Factor 7** + (herd)	5	-39	88.5	76.8	na

^a^ML logistic = multi-level binary logistic regression; Binomial logistic = Logistic regression on the proportion of positive; ML regression = multi-level regression

^b^according to Akaike Information Criterion corrected for small-sample bias (AICc)

^c^K = number of estimated parameters; logLik = log-likelihood; CV = 10-fold cross validation;

^d^(herd) = random effect variable

** p-value <0.05

* p-value <0.1

Finally, in multi-level regression on the ELISA S/P ratios, the averaged model indicated that ELISA S/P ratios were lower for migratory elk, increased with higher values of PCA factor related to ponds and small lakes (factor 7), and may decrease when cattle density on the elk home range increased, and for male elk (Table 5).

#### Faecal sample dataset

**Fig 2:**
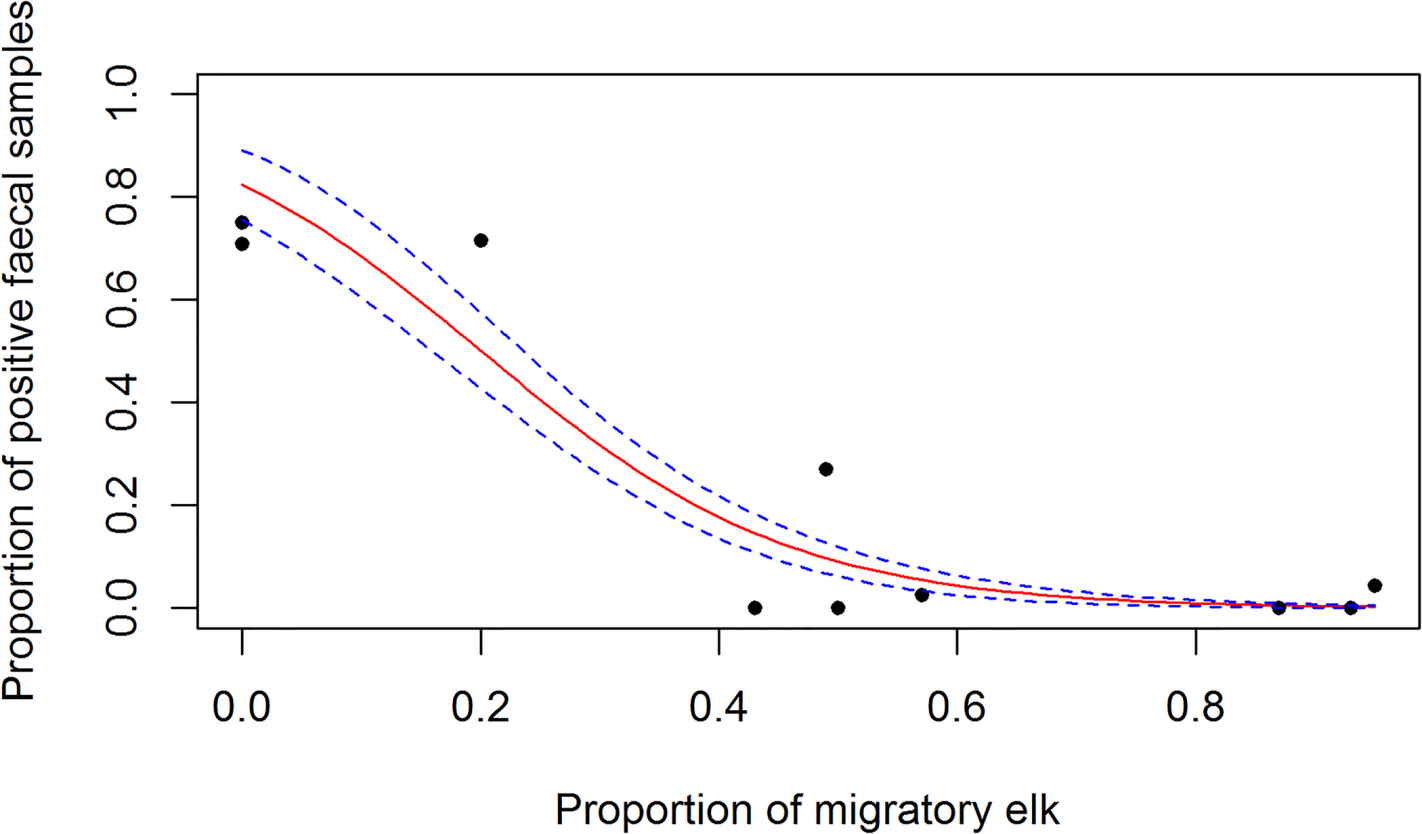
Relationship between the proportion of *F*. *magna* positive faecal samples and the proportion of migratory individuals in ten elk herds. The points represent the observed data, the red line is the fitted logistic regression and the blue lines the 95% confidence interval.

The five elk herds exposed to cattle had lower prevalence by coproscopy than the non-exposed herds (χ^2^ = 259; p<0.001) (Table 3). The herd prevalence was negatively associated with cattle density (OR = 0.26, 95% CI [0.2, 0.34]) and with the proportion of migratory individuals (OR = 0.46, 95% CI [0.41, 0.52] for a 10% increase) (Fig 2).

In multivariate analysis, the averaged model indicated a negative association between the occurrence of *F*. *magna* and the cattle density, even after inclusion of the environmental composite variables (OR = 0.23, 95% CI [0.10, 0.54]) (Table 5). The cattle density, PCA factor 2 (related to areas of higher elevation and slope with no agricultural land), and PCA factor 7 (related to ponds and small lakes) were the variables with highest relative importance and included in the best model (Table 6).

The proportion of migratory individuals in the herd was strongly negatively associated with the herd prevalence of *F*. *magna* in the multi-model averaged model (OR = 0.03, 95% CI [0.003, 0.29], along with the cattle density and the PCA factors 6 (related to open coniferous forest and water streams) (Table 5). PCA factor 2 and PCA factor 7 were positively associated with *F*. *magna* herd-level prevalence.

## Discussion

In this study, we tested the effects of migration and cattle density on liver fluke occurrence. Across models and with both the faecal sample dataset and the MERP elk datasets, we found a consistent negative relationship between migration and parasite occurrence, while the association with cattle density was stronger in the faecal sample dataset.

In our data, migratory individuals had lower risk of liver fluke infection, and if infected, had lower egg counts. This relationship was present in both datasets (faecal sample dataset, and MERP elk dataset), and was maintained in multivariate models including environmental and climate factors relevant to *F*. *magna* life cycle. Although for the field-collected faecal samples, it was not possible to determine the migratory behaviour of the elk from which faecal samples were collected, existing knowledge on the proportion of the migratory elk in each herd allowed assessing this effect at the herd level. At the herd level, *F*. *magna* prevalence decreased with the proportion of migratory individuals in the herd. The proportions of migratory elk for Jasper and Banff National Park were assumed to be low since the sampling focused on resident elk of the town sites. An increase of these proportions in the analysis did not substantially change the outcome of the model (data not shown). Observing a similar association at the herd-level and at the individual level on the MERP elk for which the migratory phenotype was known, provide strong evidence of an effect of migratory behaviour on *F*. *magna* occurrence in elk.

Altizer et al. [20] put forth two hypotheses about the relationships between migration and parasitism: the “migratory culling” and “migratory escape” hypotheses. The “migratory culling” hypothesis suggests that infected individuals may not cope with the physiologically demanding migration, and are progressively removed from the population, resulting in a lower prevalence in migratory population. Previous reports indicated a limited loss of condition of elk infected with liver fluke [38], although acute peritonitis and death following severe infections were reported [7]. It is not known if *F*. *magna* infection may increase mortality during migration or if sub-clinical effects may decrease fitness for migration, and it was not possible to explore this question further in this study. The “migratory escape” hypothesis suggests that migration away from the winter range coincides with the period of higher infection pressure [20–21]. The deposition of eggs during spring and summer results in a large number of recently encysted metacercariae on wetland vegetation in late summer and fall [7]. Hoover et al. [39] and Rognlie et al. [40] studying *Fasciola hepatica* in Idaho and Montana, respectively, also suggested that the peak of infection may not be earlier than September-October. Eggs, cercariae (in snails), and metacercariae can overwinter, however their subsequent development (in spring) is slower than eggs deposited in summer, and freezing temperatures kill eggs in advanced stage of development [22]. As a result, the first peak of infection in spring may be of lower amplitude, particularly when persistent snow cover delays emergence of snails from hibernation [7]. The average dates of departure from, and of return to, the more densely populated winter/resident home range are April 24^th^-May 6^th^ and November 13^th^- December 4^th^, respectively [24]. Thus, migratory elk may indeed escape highly contaminated ranges during their summer migration, thereby avoiding both peaks of infection.

In both datasets, there was consistently a negative association between the cattle density and the occurrence of *F*. *magna* in elk, even after adjustment for important landscape and climate factors (summarized in PCA factors) in multivariate analyses. This was consistent with the “dilution effect” hypothesis, suggesting that non-competent hosts consume infective stages, removing them from the pasture and preventing them from completing their life cycle. This “loss” of infective stage may decrease the risk of infection and the prevalence of the parasite in the competent host [13]. However, other mechanisms could be hypothesized to explain lower *F*. *magna* prevalence in higher cattle density areas. Indeed, cattle grazing along riparian corridors have been documented to affect invertebrate fauna and may result in decreasing aquatic snail density [41–42]. However, some competent *Lymnaea* species intermediate hosts show preference for habitats characterised by mud and livestock footprint [43]. It is, therefore, difficult to anticipate the effect of an increase in livestock density on aquatic snail intermediate hosts without conducting field surveys. Although anecdotal observations indicated the presence of *Lymneae* sp. in areas where very low prevalence of liver fluke infection was detected in elk (CC and BL herds), in-depth quantitative snail surveys would be necessary to identify intermediate host species, distribution, and habitat preferences in the study area. Although in this analysis, the elk migratory behaviour and the cattle density were our main two explanatory variables of interest, it is noteworthy that the importance of ponds and small lakes (PCA factor 7) is consistent with the reliance of this parasite on aquatic snails.

*Fascioloides magna* presence across the country is patchy [7], and it could be argued that our observations are a result of this patchy spatial distribution. However, these different elk herds are not in complete isolation, and inter-herd movements occur between infected and non-infected elk herds. This last interpretation would fail to explain how some herds stay non-infected despite repeated introductions of infected individuals.

The results of the serological testing of elk and cattle samples in low *F*. *magna* prevalence areas give important additional information to discuss the dilution hypothesis. Serological evidence of infection was found in elk herds with high prevalence of *F*. *magna*, but also in herds with no detectable patent infection (CC, BL, PH and WH). The telemetry data indicated that none of these seropositive individuals were dispersal elk that would have changed herd in the course of the follow-up period. This suggests that *F*. *magna* is present at low prevalence in some of these elk herds. Additionally, cattle in areas with low prevalence of elk liver fluke had evidence of exposure to *F*. *magna*. This low level circulation of *F*. *magna* in both elk and cattle supports the dilution hypothesis, rather than the mere absence of the parasite in these areas.

It was not possible to exclude the possibility of cross-reaction between the *F*. *magna* serological test and other trematodes, but with only one unconfirmed case report of *F*. *hepatica* in Alberta in 1998 [44] and the absence of other trematode eggs in the faecal samples tested (data not shown), it is not thought to compromise the validity of our results.

The potential management implications of these results are important to consider. This study suggested that, in agreement with the “migratory escape” hypothesis [20], the prevalence of *F*. *magna* was lower for elk following seasonal migratory movements. The disturbance of migratory behaviour of elk (and other wild cervids) by human land-use and activities (e.g. habitat fragmentation, provision of forage through agriculture) may have important ripple effects on the dynamic of *F*. *magna* and potentially other pathogens [45–47], by increasing the risk of infection of non-migratory individuals on highly contaminated range. These observations highlight the importance of including migratory corridors protection in land-use and land-access decisions.

Our work suggests a possible dilution effect reducing the risk of *F*. *magna* infection for elk co-grazing with non-competent cattle hosts. Interventions at the wildlife-livestock interface often involve some level of separation between domestic and wild host species (by fencing, hazing, translocation, or culling), assuming that a reduced contact between the two species will reduce the risk of pathogen transmission [48]. However, if in fact there is a dilution effect, separation of two species and homogenization of ecosystems, could result in an increase of infections for some pathogens [49]. Conservation planning and management of wildlife-livestock interfaces should take into consideration the effects of separating livestock and wild ungulate species on the existing equilibrium within their ecosystem, in particular related to pathogen dynamics.

## Supporting Information

S1 File Method of elk faecal sample collection. (DOCX)

S2 File *Fascioloides magna* antigen preparation, western blot, enzyme linked immuno sorbent assay protocols, and essay validation. (DOCX)

## Abbreviations

AICc: Akaike Information Criteria corrected for small sample bias
AUC: Area Under the Curve
BL: Beauvais Lake elk herd
BNP: Banff National Park
CC: Castle-Carbondale elk herd
CP: Crowsnest Pass
ELISA: Enzyme-Linked Immuno Sorbent Assay
FWA: Full Worm Antigens
GPS: Global-Positioning System
JNP: Jasper National Park
KMO: Keiser-Mayer-Olkin
L: Livingstone elk herd
LoCoH: Local Convex Hull
MERP: Montane Elk Research Program
OD: Optic Density
PCA: Principal Component Analysis
PH: Porcupine Hills
ROC: Receiving Operator Characteristics
Se: Sensitivity
S/P ratio: Sample-to-Positive ratio
Sp: Specificity
WH: Whaleback elk herd
TA: Tegumental Antigens
WB: Western Blot
WNP: Waterton National Park
YHT: Ya Ha Tinda
