# Supplementary material for: Better Alone or in Ill Company? The Effect of Migration and Inter-Species Comingling on Fascioloides magna Infection in Elk
Source: PLoS One. 2016 Jul 27;11(7):e0159319. doi: 10.1371/journal.pone.0159319 (PMC4963109; doi:10.1371/journal.pone.0159319)
Supplement: S1 File — (DOCX) [file pone.0159319.s001.docx]

Method of elk faecal sample collection

Faecal samples were collected from each of 9 elk herds between March 3rd, 2010 and May 15th, 2010 (while faecal samples from the Banff National Park herd were opportunistically obtained from Parks Canada wildlife officers during operations of collaring, culling or removing of road kills). Sample collection in wildlife presents some challenges due to the absence of exact census, the inability to identify individuals and, for wild cervids, the distribution of individuals into unstable sub-groups moving across a large territory. Therefore, classical randomized methods are not applicable. It has been shown that applying systematic strategies could limit the bias induced by convenience sampling of wildlife (Nusser et al., 2008). The sampling methods need to take into account the ecology and behaviour of the North American elk, the characteristics of each herd in each location and obviously the objective of the study. Sampling during winter when elk are grouped in large herds represented an optimal situation compared to summer when elk are dispersed at higher elevation. Our primary objective was the collection of fresh samples from distinct individuals, in the different sub-groups of the 10 herds.

Our strategy can be divided in 3 steps: observing, strategizing and sampling.

The observation phase aimed at identifying subgroups of a herd, observe their connection, dynamic and movement patterns. The known home range of each herd was searched based on information from local observers, knowledge extracted from GPS collar data and localization by radio frequency triangulation. To ensure that all the subgroups that were accessible at the time of sampling could be found, an intensive coverage of roads and trails was made with systematic scanning of the area by binocular observation.

After these observations, a strategy could be established. Sub-group structure changing quickly, sub-group in close proximity and highly connected were sampled the same day and would never be revisited later to avoid the resampling of individuals. In each herd, certain situations were particularly favorable to obtain fresh samples from distinct individuals. For instance, in resting areas (on snow or on the loose soil of pine forest), the oval tracks of each elk body could easily be identified and would often display a pile of pellet on one end of the oval track (Fig. S1.1). This situation would significantly reduce the risk to sampling twice the same individual. In some cases, elk were far enough apart so that the observer could wait for the animal to defecate and use landmarks to find the pellets afterward.

Finally the sample collection itself included the subgroup localization, distance observation (counting, characterization of the herd, landmark identification), approach and collection. On site, only fresh (based on consistency and color) structured pellet piles were sampled to avoid cross-contamination of samples. The top of the pile was collected with a Ziploc® bag to avoid contamination between samples and by the soil. Each sample was identified and located by GPS coordinate. A total of 616 samples were collected in the 9 elk herds included in this sampling.

The application of a systematic sampling strategy for each of the herds overcame some of the challenges associated with non-random sampling of wildlife populations. The strategy ensured the collection of fresh samples and we were confident that they were from distinct individuals in the nine elk herds.

a
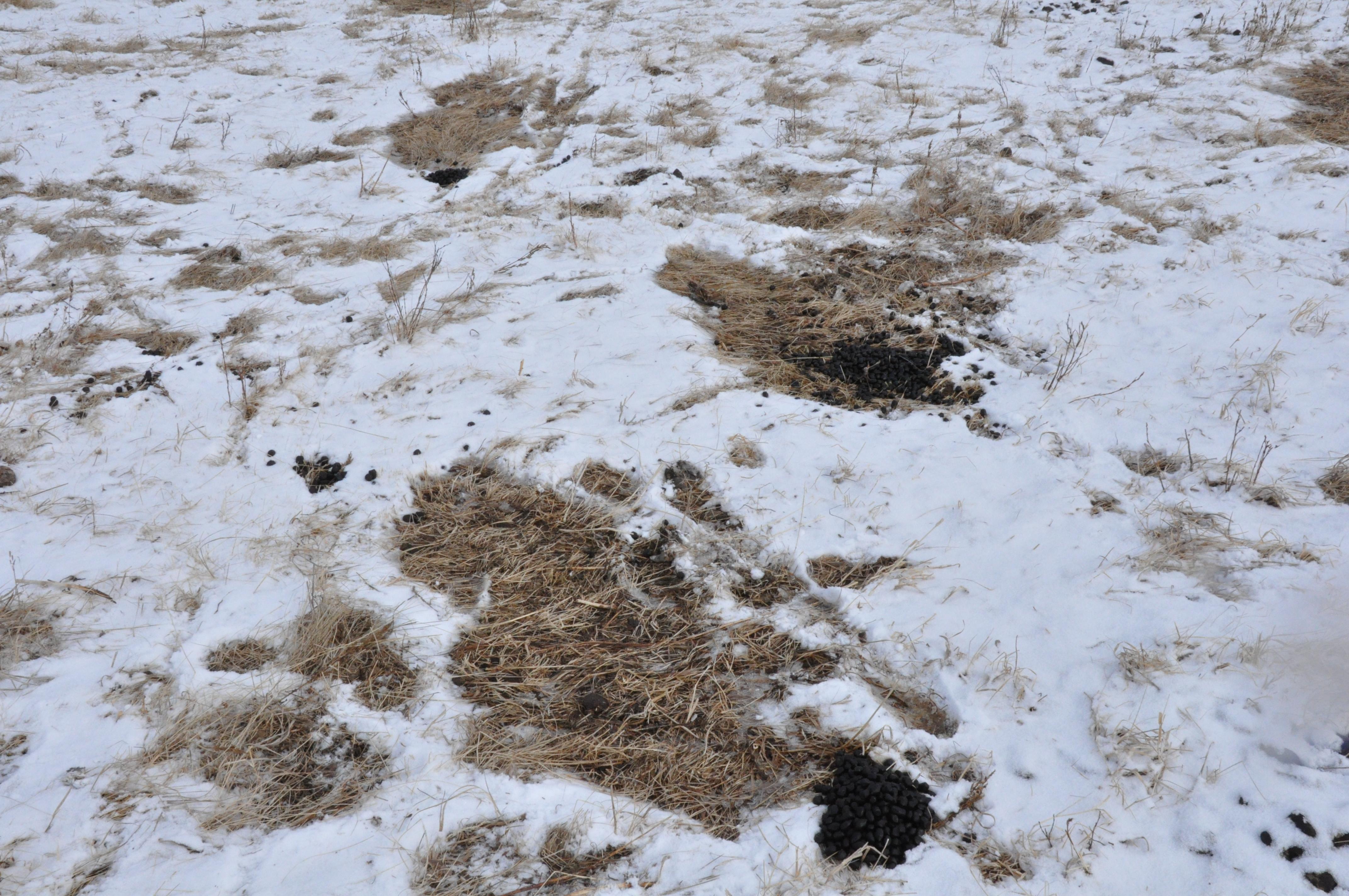

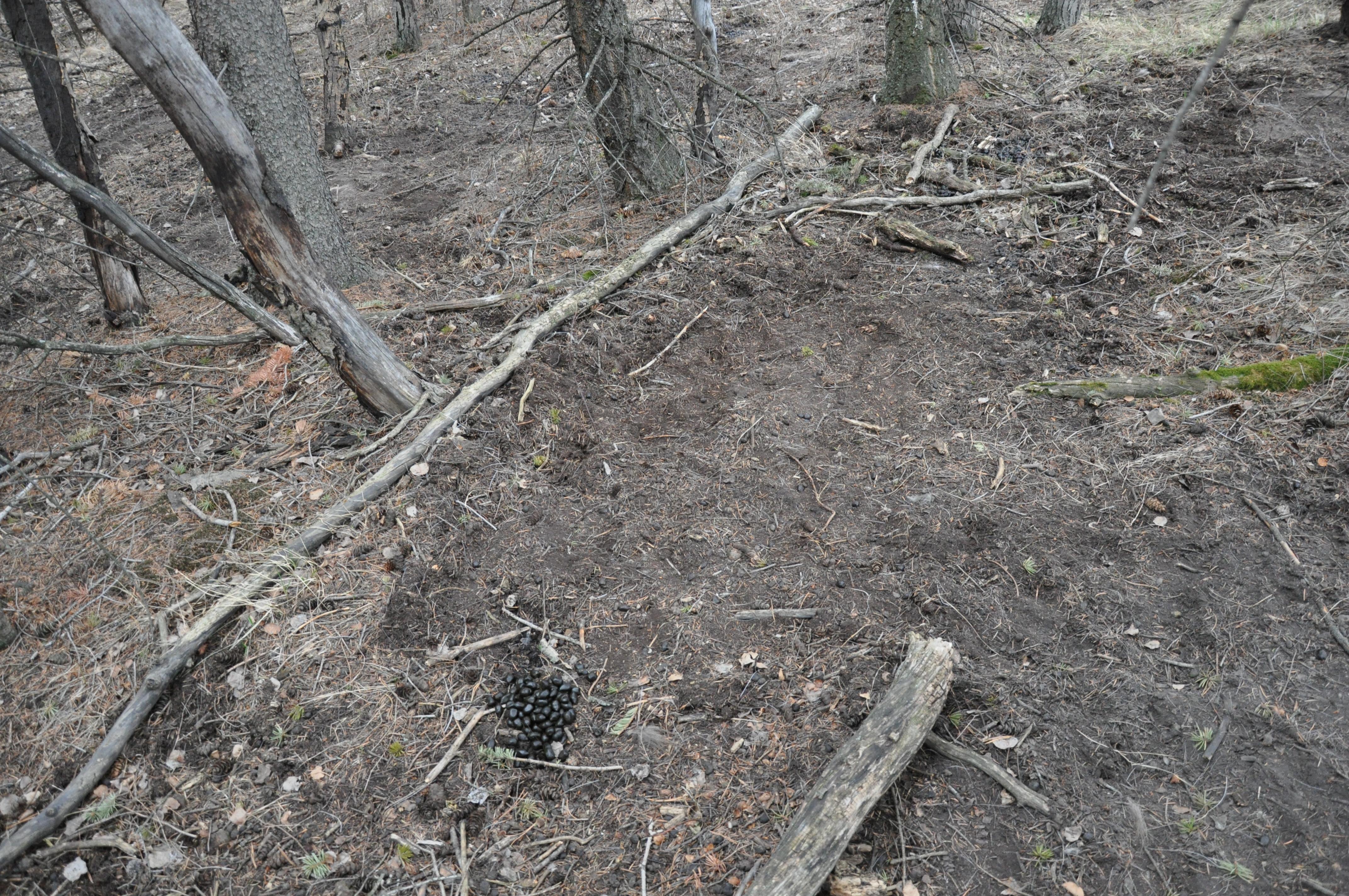
b

Fig. S1.1: Oval-shaped track of resting elk a) in snow and b) in loose soil, allowing identification of individual faecal samples.

**Reference:**

Nusser, S.M., Clark, W.R., Otis, D.L., Huang, L., 2008. Sampling Considerations for Disease Surveillance in Wildlife Populations. Journal of Wildlife Management 72, 52-60.
