# Supplementary material for: Better Alone or in Ill Company? The Effect of Migration and Inter-Species Comingling on Fascioloides magna Infection in Elk
Source: PLoS One. 2016 Jul 27;11(7):e0159319. doi: 10.1371/journal.pone.0159319 (PMC4963109; doi:10.1371/journal.pone.0159319)
Supplement: S2 File — (DOCX) [file pone.0159319.s002.docx]

***Fascioloides magna* antigen preparation, western blot, enzyme linked immuno sorbent assay protocols, and essay validation**

**Antigen collection and preparation**

Adult flukes were collected from livers of three elk obtained from Banff National Park, within 2-3 hours after the animal’s death. About 60 adult worms were collected, rinsed three times in warm sterile PBS (pH 7.2), and temporarily stored in RPMI medium 1640 (Invitrogen®) before subjecting them to antigen extraction.

Two types of antigen were prepared from the adult *F. magna* worms: full worm antigens (FWA) and tegumental antigens (TA). Extraction protocols were modified from Anuracpreeda et al.(Anuracpreeda et al., 2009). For FWA, five flukes were homogenized by sonication in 25 ml of lysis buffer (0.01M Tris-HCl, pH 7.2; 0.15M NaCl; 0.001M EDTA; 0.001M PMSF) with proteinase inhibitor cocktail (Sigma-Aldrich, USA) (200µl/10ml lysis buffer), penicillin (10000U/ml), streptomycin (10000pg/ml) and 0.5% Triton X-100 in a glass homogenizer. The homogenate was left at 4^0^C for 1 h with constant shaking. The suspension was centrifuged at 5000g for 20 min at 4^0^C and the supernatant collected. For TA, five other worms were placed in a beaker with 0.05M Tris buffer containing 0.01M EDTA, 0.15M NaCl and 1% Triton X-100 for 20 min at room temperature. The supernatant collected after centrifugation at 5000xg for 20 min at 4^0^C was further dialyzed against 0.01M PBS overnight at 4^0^C using Spectra dialysis membrane (Molecular weight cut off 6-8 KDa) (Spectrumlabs, USA). The protein concentration of the two antigen preparations was tested by Bradford protein assay (Bradford Reagent, Sigma-Aldrich, St.Louis Missouri, USA). The dialysate was lyophilized and stored at -80^0^C until further use.

**Western blot development**

The Western blot (WB) protocol was modified from (Qureshi et al., 1995). Briefly, the antigen preparations were mixed with 4X Laemelli sample buffer, incubated in a boiling water bath for 10 min, and used for blotting. For each wells, 10 microgram of total protein of antigen extract were electrophoresed in 12% SDS-polyacrylamide gels and transferred onto a nitrocellulose membrane (Bio-Rad, Hercules, CA) overnight at 4^0^C. TA antigen was loaded at 120 μg / preparative well. Membranes were blocked in 1x Western blot blocking solution (Life Technologies) for 1 h at room temperature. The blot was cut into strips, incubated individually with serum samples at a 1:50 dilution on a rocking platform at 4°C overnight. The positive control was serum from an elk confirmed infected by necropsy and coproscopy, the negative control was from a farmed elk inspected at slaughter. Blots were washed three times with TBS-T and incubated in Protein G-horseradish peroxidase (BioRad) at 1:3000 dilutions in 0.1x blocking solution for 1h at room temperature. The strips were washed twice with TBS-T and once with TBS, and then developed with membrane tetramethylbenzidine (TMB; Sigma T0565). Development was stopped by washing with distilled water. After initial optimization, the TA provided a clearer banding pattern than the FWA to discriminate positive and negative control samples, and was, therefore, used in subsequent western blot testing.

In particular, one or more of approximately 40, 37, 32, or 12 kDa bands were present in positive samples but never observed in negative samples, and were used to visually assess other samples of unknown status (Fig S2.1). We confirmed that, among elk having both serology and faecal sample results, 100% (n=15) of faecal-positive samples were also positive in WB, indicating that WB sensitivity was at least as good as the faecal egg analysis. As expected, some samples negative in coproscopic test were positive in WB (7 of 19 coproscopy-negative), possibly indicative of older or non-patent infections, intermittent shedding, or absence of eggs in the 2g of feces.

**ELISA**

Maxisorp ELISA plates (Nunc®) were coated with 50 µL of 1 g/mL FWA antigen in PBS in each well and incubated overnight at 4°C. Plates were washed 3 times with a wash solution (PBS containing 0.1% Tween20, hereafter referred to as PBS-T), then blocked with 300µl PBS solution with 1% BSA and 1% Tween20, for 2 hours at room temperature. For each animal, 100 µL of 1:100 serum in 0.1% BSA PBS-T was added to each well in duplicate and incubated at 37°C for 1 hours.  After washing three times, plates were incubated for 1 hour at 37°C with 50 µl of Protein G - Horseradish Peroxidase conjugate (PG-HRP; Biorad) at a 1:5000 dilution in 0.1% BSA PBS-T.  Plates were washed three times, and revealed with 50µl Tetramethyl benzidine substrate (TMB) (Sigma T4444) and stopped with 50µL H_2_SO_4_ (2N) solution (Sigma S5814) when the colour of the positive control samples reached a visually estimated optic density OD=1.  The OD of each well was measured using a spectrophotometer at 450 nm wavelengths (PE Victor X3 plate reader).

**ELISA validation**

A combination of coproscopic results and WB was used in order to constitute panels of elk *F. magna*-positive and –negative control samples. Samples were classified as negative if both coproscopic and WB tests were negative, and positive if at least one of the tests was positive. All MERP elk sera were tested by ELISA based on the FWA: for each well, the optic density (OD) value was re-expressed as a sample-to-positive ratio (S/P ratio = [OD_Sample_-OD_Neg_]/[OD_Pos_-OD_Neg_]) based on a positive and negative reference samples selected during preliminary testing and consistently used across all plates. A Receiving Operator Characteristics (ROC) analysis was then performed in the R package pROC (Robin et al., 2011), to identify the optimal cut-off for the ELISA and estimate sensitivity (Se) and specificity (Sp). For cattle serum samples, the ELISA was used as a screening test and only samples with S/P ratios higher than 0.65 (using a positive serum sample from previously confirmed infected cow as reference) were processed in WB. To ensure the screening procedure had an appropriate sensitivity, we additionally tested 30 samples with S/P ratios lower than 0.65.

Using a combination of flukefinder coproscopy and western blot, we obtained a pool of 28 positive and 72 negative samples that we used for the ELISA evaluation. The distribution of ELISA S/P ratios for positive and negative panel samples is illustrated in Fig S2.2a. The ROC curve indicated an area under the curve of 0.98, and that both sensitivity and specificity were optimized for a S/P ratio cut-off value of 0.7 (Fig S2.2b). In this case, to reduce the risk of false positive, we selected a cut-off of 0.9 that optimizes the specificity (99%, 95% Confidence Interval (CI) [93, 100]) while keeping an acceptable sensitivity (89%, 95% CI [72, 98]) (Fig S2.2b).

In cattle, the ELISA was used as a screening test, and among the 57 cattle sera with ELISA S/P ratio above 0.65 and tested in western blot, 20 were WB-positive. The 30 samples with ELISA S/P ratios below 0.65 were all WB-negative.

**Fig. S2.1. Western blot of 2 positive elk sera (+) and 2 negative elk control sera (-)**

The arrow indicates the ~40, 37, 32 and 12 kDA band used to discriminate positive and negative samples in subsequent testing


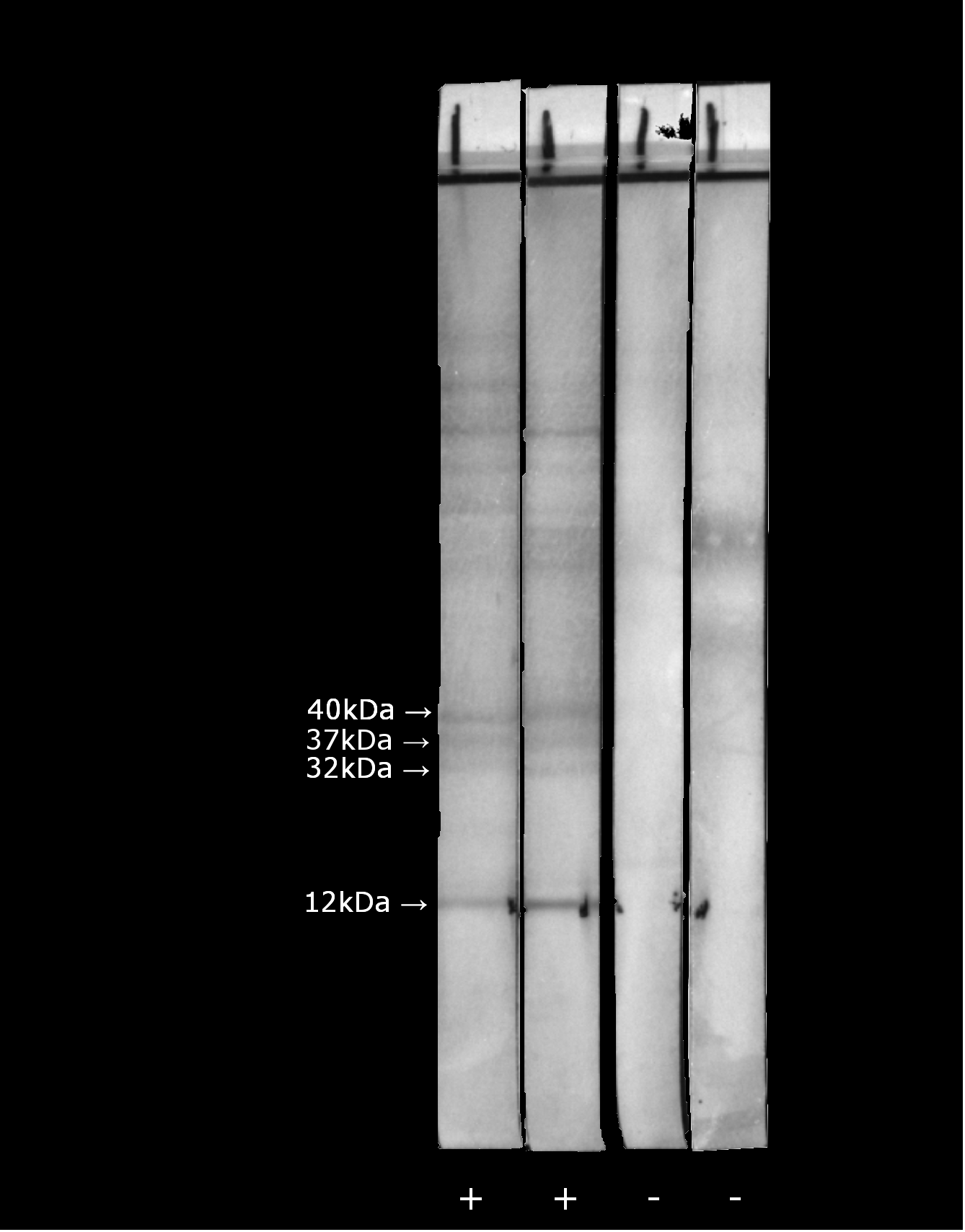


**Fig S2.2. Development and evaluation of *Fascioloides magna* ELISA.** a) Distribution of ELISA S/P ratios in panels of *Fascioloides magna* positive and negative elk sera: Red and blue bars indicate the S/P ratios of positive and negative sera, respectively. The overlap of the two distributions appears in purple; b) Receiving Operator Characteristic (ROC) curve of the ELISA tested with panels of 28 positive and 72 negative elk serum samples: the label of the points on the ROC curve (e.g. 0.9 (98.6%, 89.3%)) indicate: S/P ratio cut-off (Specificity, Sensitivity); AUC=Area Under the Curve


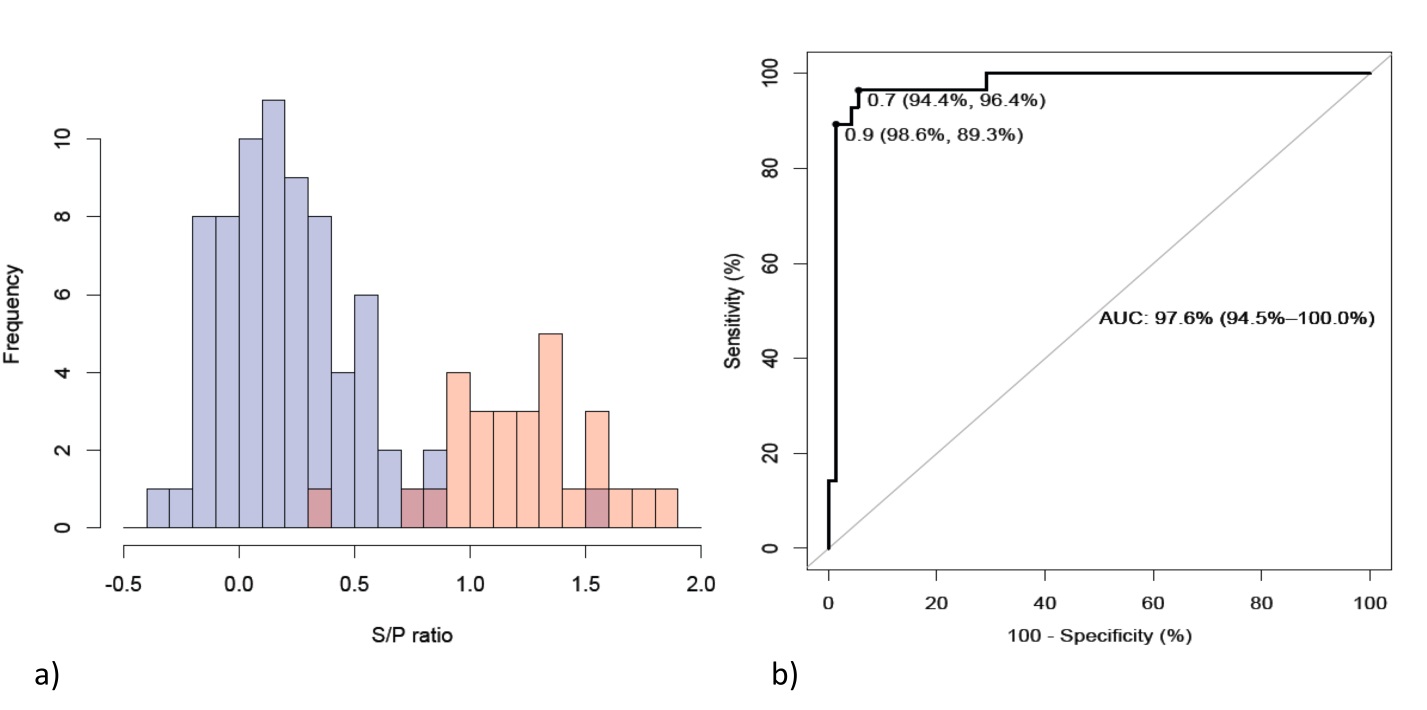


**References**

Anuracpreeda, P., Wanichanon, C., Chawengkirtikul, R., Chaithirayanon, K., Sobhon, P., 2009. Fasciola gigantica: immunodiagnosis of fasciolosis by detection of circulating 28.5 kDa tegumental antigen. Experimental parasitology 123, 334-340.

Qureshi, T., Wagner, G.G., Drawe, D.L., Davis, D.S., Craig, T.M., 1995. Enzyme-linked immunoelectrotransfer blot analysis of excretory-secretory proteins of Fascioloides magna and Fasciola hepatica. Vet Parasitol 58, 357-363.

Robin X, Turck N, Hainard A, Tiberti N, Lisacek F, Sanchez JC, Müller M. pROC: an open-source package for R and S+ to analyze and compare ROC curves. BMC Bioinformatics 2011; 12: 77. DOI: 10.1186/1471-2105-12-77
